# Remaining Useful Life Prediction for Lithium-Ion Batteries Based on Gaussian Processes Mixture

**DOI:** 10.1371/journal.pone.0163004

**Published:** 2016-09-15

**Authors:** Lingling Li, Pengchong Wang, Kuei-Hsiang Chao, Yatong Zhou, Yang Xie

**Affiliations:** 1 Province-ministry Joint Key Laboratory of Electromagnetic Field and Electrical Apparatus Reliability, Hebei University of Technology, Tianjin, 300130, China; 2 College of Electrical Engineering and Computer Science, National Chin-Yi University of Technology, Taichung, 41170, Taiwan; 3 School of Electronic and Information Engineering, Hebei University of Technology, Tianjin, 300130, China; Beijing University of Technology, CHINA

## Abstract

The remaining useful life (RUL) prediction of Lithium-ion batteries is closely related to the capacity degeneration trajectories. Due to the self-charging and the capacity regeneration, the trajectories have the property of multimodality. Traditional prediction models such as the support vector machines (SVM) or the Gaussian Process regression (GPR) cannot accurately characterize this multimodality. This paper proposes a novel RUL prediction method based on the Gaussian Process Mixture (GPM). It can process multimodality by fitting different segments of trajectories with different GPR models separately, such that the tiny differences among these segments can be revealed. The method is demonstrated to be effective for prediction by the excellent predictive result of the experiments on the two commercial and chargeable Type 1850 Lithium-ion batteries, provided by NASA. The performance comparison among the models illustrates that the GPM is more accurate than the SVM and the GPR. In addition, GPM can yield the predictive confidence interval, which makes the prediction more reliable than that of traditional models.

## 1. Introduction

Lithium-ion batteries are widely used in electric cars, portable terminals, and military devices. As a complex electrochemistry system, Lithium-ion batteries will gradually degenerate or even fail with time, leading to the machine halt and even serious accidents. As a result, prediction of the Remaining Useful Life (RUL) of Lithium-ion batteries is of great importance to guarantee devices safe and stable [1]. RUL can be measured by the number of charge and discharge cycles for the given battery to reduce its capacity from the known current value to the threshold value [2]. An effective method to predict the RUL of a Lithium-ion battery is to estimate the properties of its capacity and the state of charge and discharge with the given failure threshold [3].

Specifically, the existing ways to predict the RUL are classified to two categories: physical models and statistical models. Physical models simulate the physical and chemistry process of the degeneration [4]. Since the physical and chemistry process of the charge and discharge is complicated, it is rather difficult to establish a physical model for RUL. Existing methods to approximately fit the degenerate trajectory include particle filter [5], Dempster-Shafer theory [6], and Grey theory framework [7], and recursive Bayesian method [8], etc. However, these methods cannot accurately fit the RUL of a real Lithium-ion battery, since the degenerate trajectory is largely influenced by the surrounding environment and the loading condition.

In contrast, the statistical models, including Artificial Neural Network (ANN) [9], Autoregression model [10], Support Vector Machine (SVM) [11–12], and Relevance Vector Machine (RVM) [13], etc. are more feasible. These models have satisfactory predictive performance in usual cases, but each of them has its own disadvantage. For example, it is difficult to choose the topological structure and initial values of parameters for the ANN, whereas the convergence rate heavily relies on the initial values. The Autoregression model needs offline training with data from a large number of Lithium-ion batteries. The SVM cannot provide probability information like confidence level. The RVM can provide probability information but has slow training speed and poor prediction in the long term.

In recent years, Goebel et al. [3] and D. T. Liu et al. [14] have achieve excellent performance in predicting the RUL by adopting the Gaussian Process Regression (GPR) model to fit the capacity curve of Lithium-ion batteries. The capacity of battery is the charge that battery can hole or release, which described by *Ah* or *mAh*. The capacity curve is made up of battery capacity value according to the battery charge and discharge cycle. The GPR model puts the priors directly on the function to be fit and thus can output probability information. Besides, the training of the GPR model is simple and efficient with matrix computation. However, since Lithium-ion batteries have properties like the self-charge and the capacity regeneration, their capacity curve is non-linear and unstable rather than smooth and purely decreasing. Particularly, the self-charge can result in large fluctuation of capacity curves, which divides the curves into multiple segments with different properties. Hence, capacity curves have multimodality that cannot be accurately described by a single GPR model. Gaussian Process Mixture (GPM) model can process it by fitting different segments with different GPR models separately, such that the tiny differences among the periods are revealed. Hence, GPM model is better than a single GPR model for the prediction of the remaining life of Lithium-ion batteries. More importantly, compared with non-probabilisitc models like SVM and ANN (e.g. RBF network), GPM model can generate the predictive confidence interval and confidence level. And the reliability of the predicted results can be measured by confidence interval.

Since the GPM model combines the advantages of the GPR, the SVM and the RBF model, and avoids their disadvantages, we propose an efficient hard-cut EM algorithm for GPM model, and then apply GPM to the prediction of the remaining life of Lithium-ion batteries. The GPM model is demonstrated to be effective for prediction by the excellent predictive result of the experiments on the two commercial and chargeable Type 1850 Lithium-ion batteries, provided by the US National Aeronautics and Space Administration (NASA).

## 2. Gaussian Processes Mixtures

### 2.1 Gaussian processes mixtures: an overview

The Gaussian Process is an essential stochastic process with excellent properties like infinitely mean square differentiable. The mixture model can be mathematically defined as following:

$${\left[y_{1},y_{2},\cdots ,y_{N}\right]}^{T}\sim N\left({\left[\mu (x_{1}),\mu (x_{2}),\cdots ,\mu (x_{N})\right]}^{T},{\left[K(x_{i},x_{j})\right]}_{N\times N}+\sigma^{2}I_{N}\right),$$ (1)

where **x**_*i*_ ∈ *R*^*d*^ and *y*_*i*_ ∈ *R* are the input and target of the i-th training sample, respectively, *μ*(•) and *K*(•, •) denote the mean function and covariance function respectively, *σ*^2^ denotes the noise variance, and *I*_*N*_ is an N-order identity matrix. In most cases, the zero mean function and Gaussian covariance function are adopted, that is

μ(x)≡0, (2)

$$K(x_{i},x_{j})=l^{2}\text{exp}\left(-{\left\|x_{i}-x_{j}\right\|}^{2}/2\delta^{2}\right),$$ (3)

where *l* and *δ* are the kernel parameters to be estimated. The most commonly used algorithm for parameter learning is maximum likelihood estimation (MLE), given by

$$(l,\delta ,\sigma )=\underset{l,f,\sigma }{\text{arg max}}\;N\left(Y|0,{\left[l^{2}\text{exp}(-{\left\|x_{i}-x_{j}\right\|}^{2}/2\delta^{2})\right]}_{N\times N}+\sigma^{2}I_{N}\right)$$ (4)

After parameter learning, given a test input x*, the predictive distribution of the test target y* is derived by Bayesian formula:

$$y*|x*\sim N\left[K(x*,X){\left[K(X,X)+I_{N}\right]}^{-1}Y,K(x*,x*)-K(x*,X){\left[K(X,X)+I_{N}\right]}^{-1}K(X,x*)\right]$$ (5)

where **X** = [**x**_1,_ **x**_2, ⋯,_ **x**_*N*_]^*T*^, **Y** = [*y*_1,_ *y*_2, ⋯,_ *y*_*N*_]^*T*^, and the kernel matrices are denoted as

$$K(x*,X)={\left[K(x*,x_{j})\right]}_{1\times N}$$

$$K(X,x*)=K{\left(x*,X\right)}^{T}={\left[K(x_{i},x*)\right]}_{N\times 1},$$ (6)

$$K(X,X)={\left[K(x_{i},x_{j})\right]}_{N\times N}$$

Generally, the mean *K*(x*, **X**)[*K*(**X**, **X**) + **I**_*N*_]^−1^**Y** is taken as the predictive value of y*.

GPM can be seen as a special mixture of experts (ME) model where each expert is a GPR model. Like ME model, we denote *z*_*i*_ = *c* if the i-th sample belongs to the c-th GPR expert, and then denote **X**_*c*_ = {**x**_*i*_: *z*_*i*_ = *c*} and **Y**_*c*_ = {**y**_*i*_: *z*_*i*_ = *c*} as the inputs and the corresponding targets in the c-th GPR, respectively. The GPM model is thus mathematically defined as

$$Y_{c}|X_{c}\sim N\left(\mu_{c}(X_{c}),K_{c}(X_{c},X_{c})+\sigma_{c}^{2}I_{N}^{c}\right),$$ (7)

where *μ*_*c*_(•) and *K*_*c*_(•, •) are the mean function and covariance function of the c-th GPR, respectively, and $\sigma_{c}^{2}$ is the variance of noise of the c-th GPR.

There are various forms of GPM model. According to the way in which the dataset is generated, GPM model can be classified into the discriminative model where inputs are fixed [15–17] and the generative model where the inputs are random vectors [18–20]. The gating function, defined as the distribution of the partition of the samples, can be fixed mixing proportions [15, 18], Dirichlet Process [19, 20], Pitman-Yor Process [16], etc. Besides, in some GPM models, various priors can be imposed onto the model parameters.

Most recently, we constructed a simplified and refined GPM model [18], where we adopted fixed mixing proportions as the gating function:

$$P(z_{i}=c)=\pi_{c}$$ (8)

The inputs in each GPR expert are independent and subjected to Gaussian distribution as follows

$$\left(x_{i}|z_{i}=c\right)\sim N(\mu_{c},S_{c})$$ (9)

Then, given the indicators *z*_*i*_ and the inputs *x*_*i*_, the outputs *y*_*i*_ are generated via Eq (7), where we set *μ*_*c*_ ≡ 0, and adopt Gaussian covariance function:

$$K_{c}(x_{i},x_{j})=l_{c}^{2}\text{exp}\left(-{\left\|x_{i}-x_{j}\right\|}^{2}/2\delta_{c}^{2}\right)$$ (10)

where the heterogeneity among each GPR is seen in the parameters *μ*_*c*_, **S**_*c*_, $l_{c}^{2}$, $\delta_{c}^{2}$, $\sigma_{c}^{2}$ with different values of *c*.

### 2.2 Proposed hard-cut EM training algorithm

However, the original EM algorithm for MGP model has exponential time complexity [18]. In order to reduce the computational burden, we propose an efficient hard-cut EM algorithm, which assumed the posterior of the indicators *z*_*i*_ to be 0 and 1. More specifically, the partition was determined via the maximum posterior criterion in each iteration. In this way, M step has been simplified into the parameter training of each GPR expert via MLE, independently. The procedure of this hard-cut EM algorithm is shown below:

Step1: Partition the training samples ${\left\{\left(x_{i},y_{i}\right)\right\}}_{i=1}^{N}$ into C groups via clustering algorithms such as k-means.
Step2 (M-step): Learn the parameters of each GPR via MLE independently, in which the mixing proportions *π*_*c*_ and input parameters *μ*_*c*_ and *S*_*c*_ has the following closed form estimation:
  $$\pi_{c}\leftarrow \frac{1}{N}\sum_{i:z_{i}=c}1$$ (11)
  $$\mu_{c}\leftarrow \sum_{i:z_{i}=c}x_{i}/\sum_{i:z_{i}=c}1$$ (12)
  $$S_{c}\leftarrow \sum_{i:z_{i}=c}(x_{i}-\mu_{c}){\left(x_{i}-\mu_{c}\right)}^{T}/\sum_{i:z_{i}=c}1$$ (13)
Step3 (E-step): partition the training samples by maximizing the posterior of the partition, that is,
  $$z_{t}\leftarrow \underset{1\leq c\leq C}{\text{arg max}}\;P(z_{t}=c|x_{t},y_{t})=\underset{1\leq c\leq C}{\text{arg max}}\;\pi_{c}N(x_{t}|\mu_{c},S_{c})N(y_{t}|0,l_{c}^{2}+\sigma_{c}^{2})$$ (14)

If the partition remains the same as the previous iteration, the training algorithm terminates; Otherwise, return to Step2.

After the training process, the partition of the test input x* can be given similarly by maximizing the posterior:

$$z=\underset{1\leq c\leq C}{\text{arg max}}\;P(z*=c|x*)=\underset{1\leq c\leq C}{\text{arg max}}\;\pi_{c}N(x*|\mu_{c},S_{c}),$$ (15)

Since the partition of all the training and test samples are given, the predictive distribution of each test sample can be obtained by applying Eq (7) to each step.

## 3. Residual Useful Life Prediction Based on GPM

### 3.1 NASA Lithium-ion batteries RUL dataset

In this paper, a dataset of four commercial Type 1850 Lithium-ion batteries from the Prognostics Center of Excellence (PCoE), NASA [21] is used. These batteries are numbered No.5, No.6, No.7 and No.18, respectively, and their Capacity ratings are all 2.0Ahr. In the experiment, these batteries repeatedly underwent charge, discharge and impedance measure in the room temperature (25°C). Specifically:

1. Charging: Charge the batteries with constant electric current 1.5A until the voltage reached 4.2V. Then, charge them with constant voltage until the charging current decreased to 20mA. Stop charging.
2. Discharging: Discharge the 4 batteries with 2A current until their voltage declined to around 2.7V, 2.5V, 2.2V, 2.5V, respectively.
3. Impedance measuring: Record the batteries impedance with the electrochemical impedance spectroscopy survey meter between 0.1Hz and 5kHz. The batteries gradually degenerated with the repeated cycles above. We use capacity to describe the property of the batteries. The experiment terminated when the capacities of the batteries declined to about 70% of their capacity ratings (the given failure threshold), since the batteries were thought to expire at that time.

**Fig 1:**
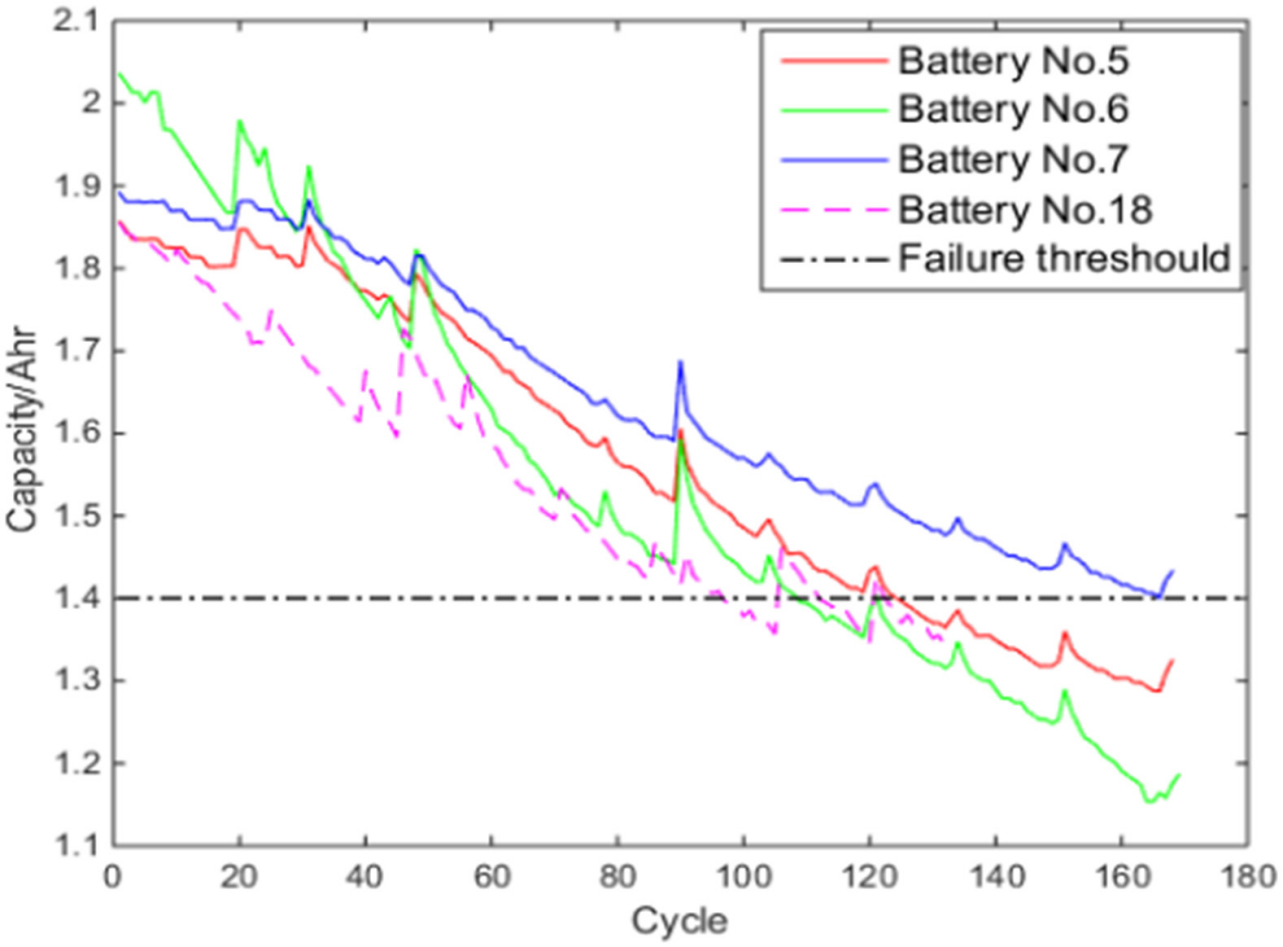
Capacity degeneration trajectories of four batteries.

Fig 1 shows the capacity degeneration trajectories of the four batteries, where the black dot line is the given failure threshold. It can be observed that there are multiple variations of trajectories, which divided the capacity curves into segments with different properties. And there are large discrepancy among the degenerate trajectories and the useful life of four batteries.

**Fig 2:**
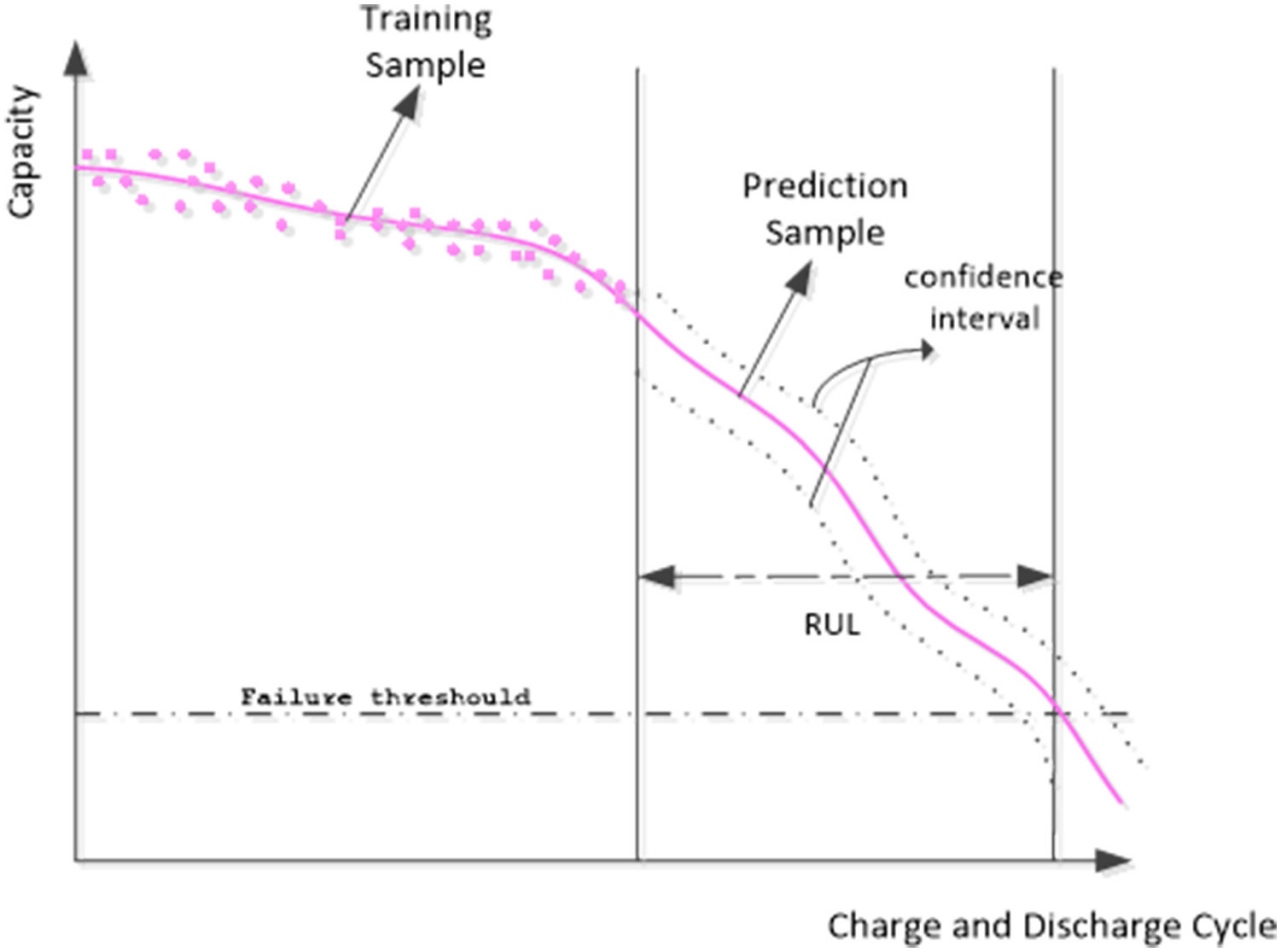
Prediction sketch of the RUL of batteries.

Fig 2 shows the prediction sketch of the RUL of these batteries, where the x-axis and the y-axis are the charge and discharge cycle and the current capacity of the batteries, respectively. As shown in Fig 2, the training sample set was constructed from the capacity trajectories data. Then, the GPM model learns the underlying dependence that is hidden in the limited number of training samples. Once such dependence has been accurately estimated, the future charge and discharge cycle could be predicted based on this dependency. Finally, RUL was obtained from the prediction result and the failure threshold. It can be concluded from Fig 2 that the GPM model can output the predictive confidence interval, which is used to measure the confidence level of prediction.

### 3.2 Prediction based on GPM and Takens’ theorem

The capacity trajectories data of Lithium-ion batteries can be seen as time series [22–23]. For sake of convenience, we denote the time series ${\left\{s\left(n\right)\right\}}_{n=1}^{\infty }$. It is defined as a function *s* of an independent variable *n*. Since the underlying dependency cannot be described exactly, a typical approach is to predict time series’ future behavior by constructing a prediction model which takes into account the observation of past values. Takens’ theorem ensures reliability of this kind of prediction [24]. To be more specific, assume that exists a smooth map *f*: *R*^*d*^ → *R* in the reconstruction phase space such that

s(n)=f[s(n−1),s(n−2),⋯,s(n−d)], (16)

If the map *f* were known, the value of series *s* at *n* is uniquely determined by its *d* values in the past. So the prediction can be achieved by estimating the map *f*.

For simplicity of notation, we define the scalar *y*_*n*_ ≡ *s*(*n*) and the *d*-dimensional vector **x**_*n*_ ≡ (*s*(*n*−1), *s*(*n*−2), ⋯, *s*(*n*−*d*))^*T*^ in such a way that Eq (16) can be written simply as

$$y_{n}=f\left(x_{n}\right)$$ (17)

In order to estimate *f*, a training samples set *D*_*N*_ with capability *N* can be constructed as follows:

$$D_{N}=\;\{\left(x_{n},t_{n}\right)\in R^{d}\times R|n=\;1,\cdots ,N\}.$$

The choice of the embedding dimension *d* and time delay *τ* are crucial to prediction. The traditional methods, such as false nearest neighbor [25], delayed mutual information, and Cao’s method [26]. To acquire better performance, we vary the combination (*d*, *τ*) through a wide range [27–28]. For the given GPM model, the dependence of root mean square error (RMSE) on (*d*, *τ*) can be obtained. After that, the combination that minimizes RMSE is selected for the optimal value. RMSE is defined by

$$R_{MSE}=\sqrt{\frac{1}{N}\sum_{n=1}^{N}{\left(y_{n}-{\hat{y}}_{n}\right)}^{2}}$$ (18)

where *y*_*n*_ and ${\hat{y}}_{n}$ denote the true target value and prediction value of the *i*-th test sample respectively.

**Fig 3:**
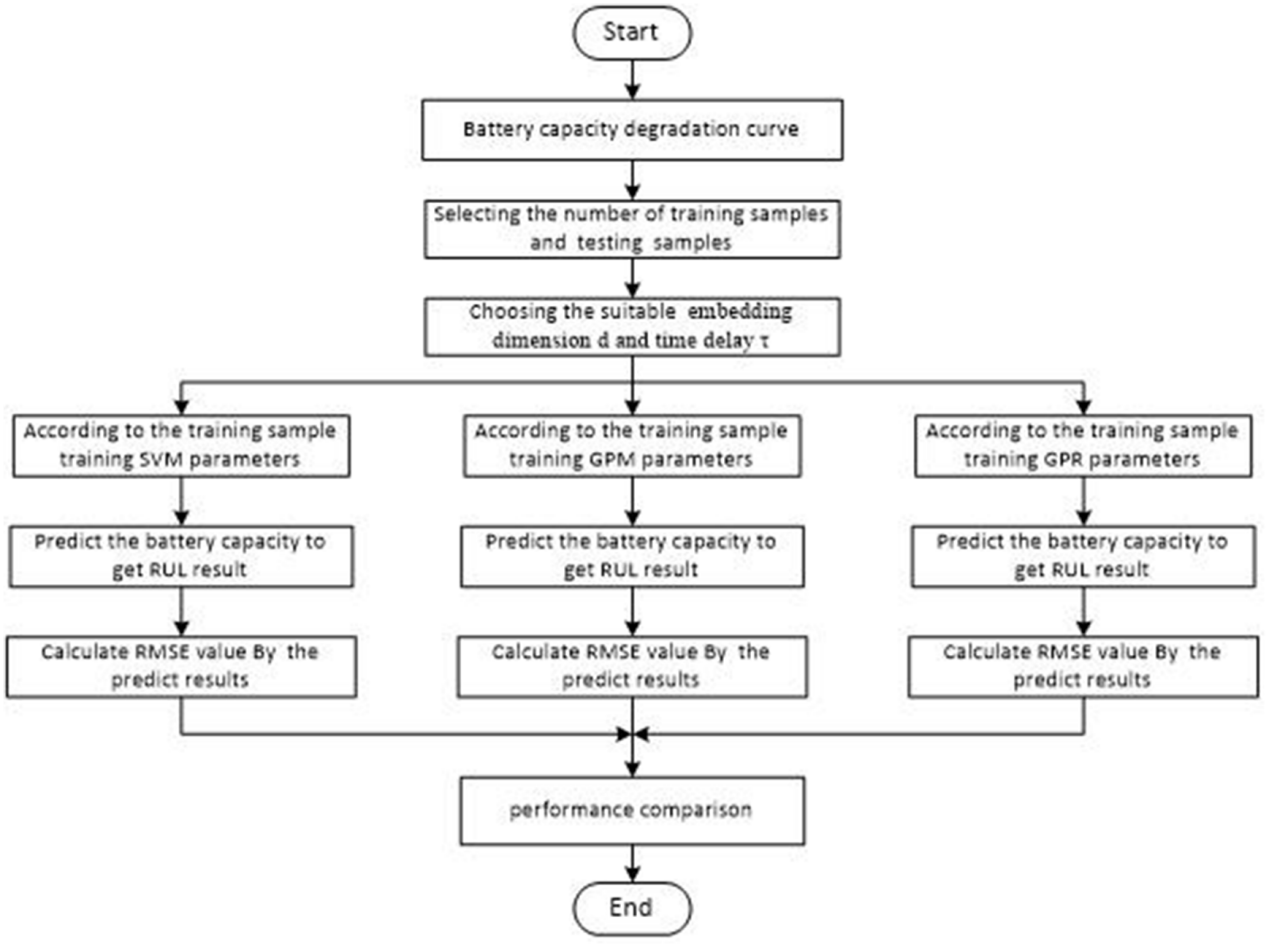
Overall technical roadmap for battery RUL predict.

Fig 3 shows overall technical roadmap for battery RUL prediction; where given three algorithms SVM, GPR and GPM for the process RUL predict. Details will be given in the fourth part.

## 4. Experiments

**Fig 4:**
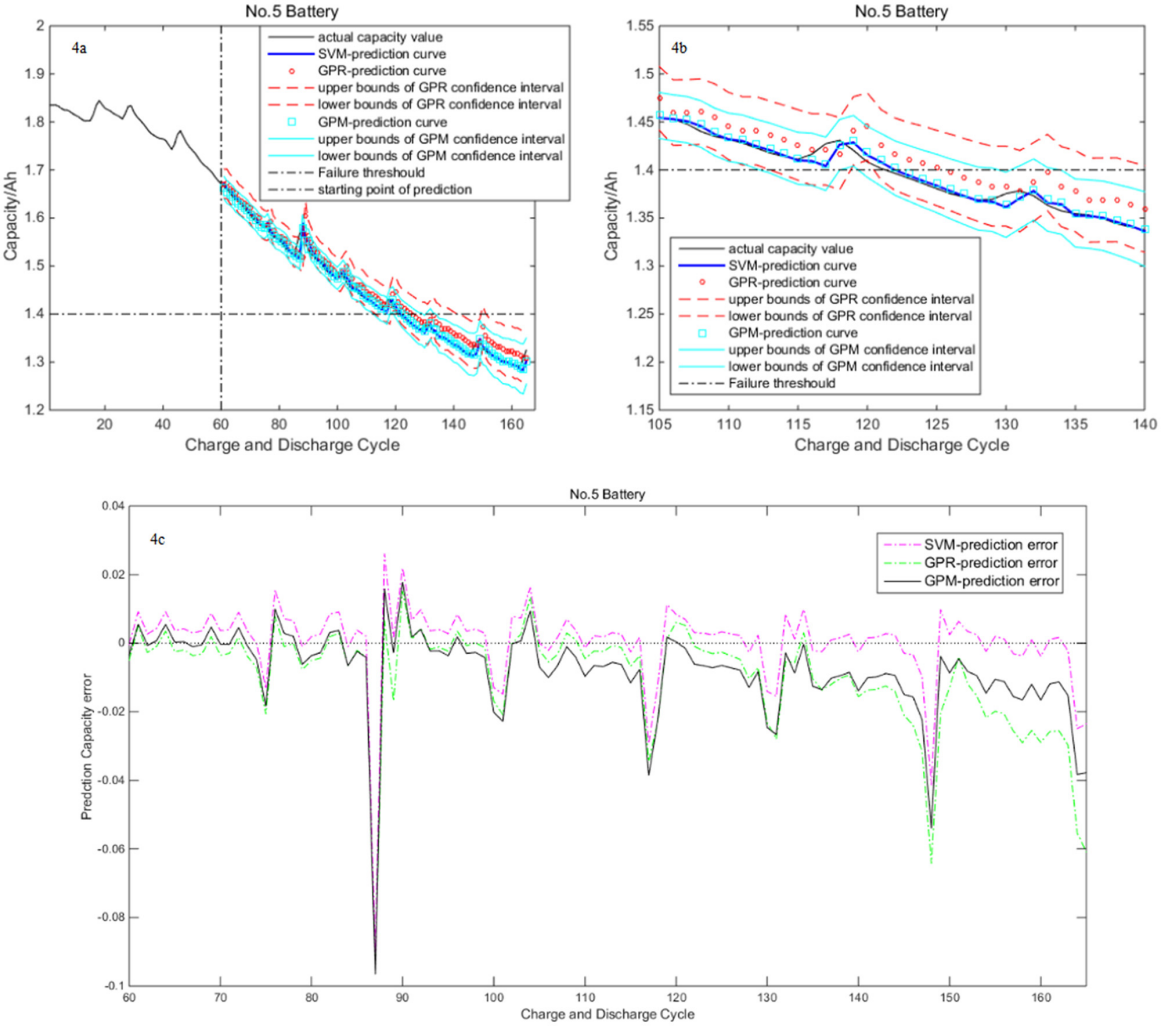
Prediction results of No.5 battery with the 60^th^ cycle as the prediction starting point. (a) Prediction result of three models. (b) Zoomed section near failure threshold. (c) Prediction error curve of three models.

In the first part, we choose No.5 battery to predict it’s RUL. Three models include SVM, GPR and GPM are used to do RUL prediction by using the same parameters based on the Takens’ theorem and phase space reconstruction. Fig 4 shows the Lithium-ion battery RUL prediction results by fixing the 60^th^ charge and discharge cycle as the prediction starting point. It means that the former 60 cycle data are chosen to construct learning samples. These samples are used to train model. The rest of cycle data are chosen to construct testing samples.

The whole experiments are operated on the Lenovo brand laptops by Intel Core i5 1.6 GHz CPU and 4 GB Memory. The software platform is the Matlab R2014b.

Fig 4(a) shows the capacity prediction values of three models. The prediction confidence interval of GPR and GPM is also shown in the figure. In order to clearly demonstrate the relationship among practical capacity, prediction capacity and the confidence interval of the prediction capacity, zoomed section near the failure threshold is shown in Fig 4(b). As seen in the figure, three models can satisfactorily predict the trends of Lithium-ion battery capacity degradation. We notice that the prediction accuracy of SVM is slightly better than GPM, and GPR have the worst results, but the SVM can't output prediction confidence interval. GPM’s prediction is more accurate than GPR. Furthermore, its confidence interval’s width is less than the confidence interval of GPR. This means GPM’s prediction is more credible than GPR. Fig 4(c) exhibits prediction error curve of three models. It is found that all the prediction errors are not more than 0.1. GPR owns a bigger prediction error. The SVM range of error margin is slightly better than GPM.

**Fig 5:**
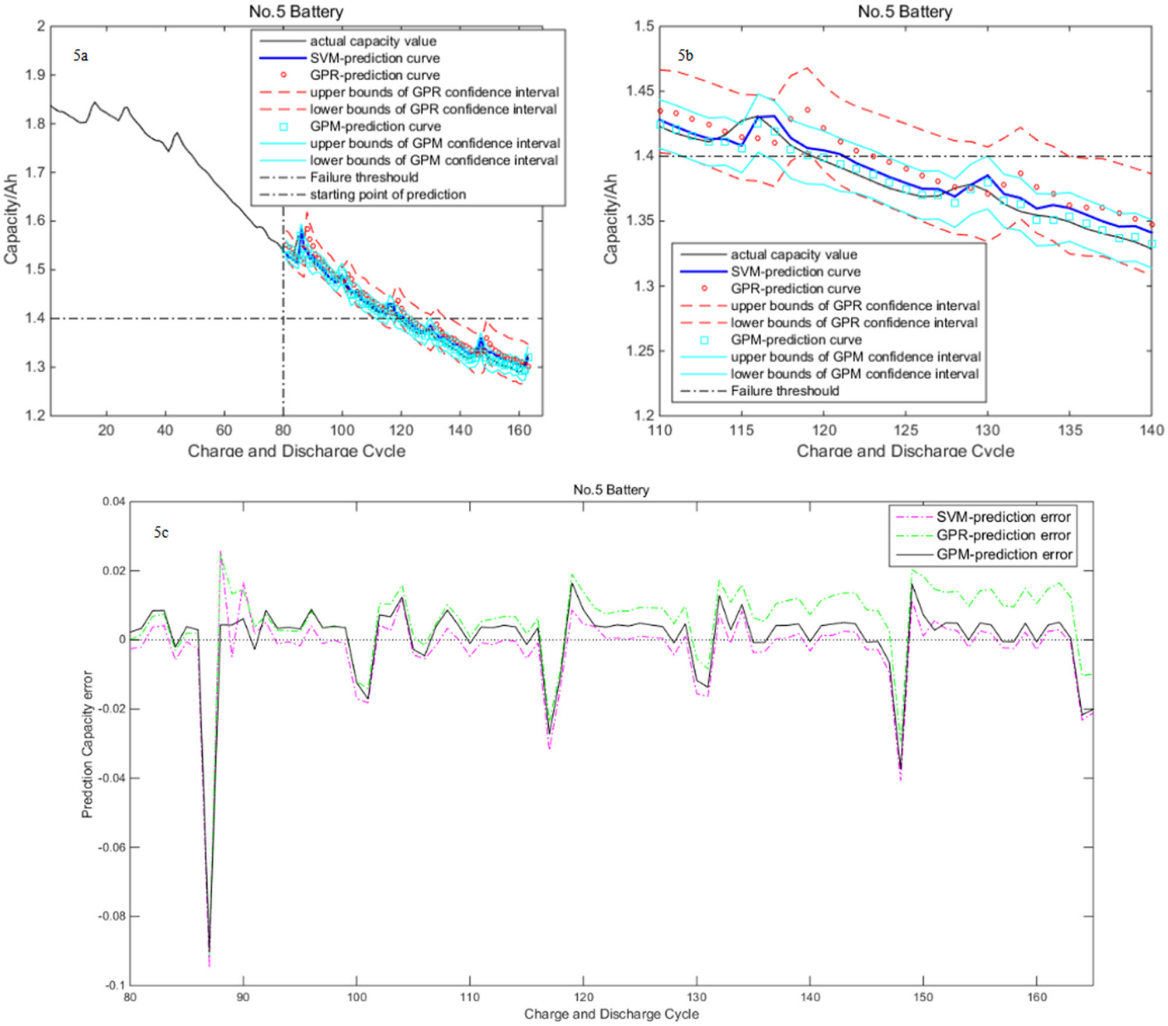
Prediction results of No.5 battery with the 80^th^ cycle as the prediction starting point. (a) Prediction result of three models. (b) Zoomed section near failure threshold. (c) Prediction error curve by three models.

Fig 5 shows the prediction results of No.5 battery with the 80^th^ cycle as the prediction starting point. The former 80 cycle data are chosen to construct learning samples, and the rest of cycle data are chosen to construct testing samples. Compared to Fig 4, the number of training samples has increased in Fig 5. As seen in Fig 5(a) and 5(b), GPM have competitive prediction accuracy with SVM, while GPM have better prediction accuracy than GPR and smaller confidence interval width. Thus is complying with the results revealed by Fig 4(a) and 4(b). By comparing Figs 4(c) to 5(c), we notice that the up bound of prediction error has decreased from 0.1 to 0.09, which means the three models’ prediction accuracy have been improved by increasing learning samples.

**Fig 6:**
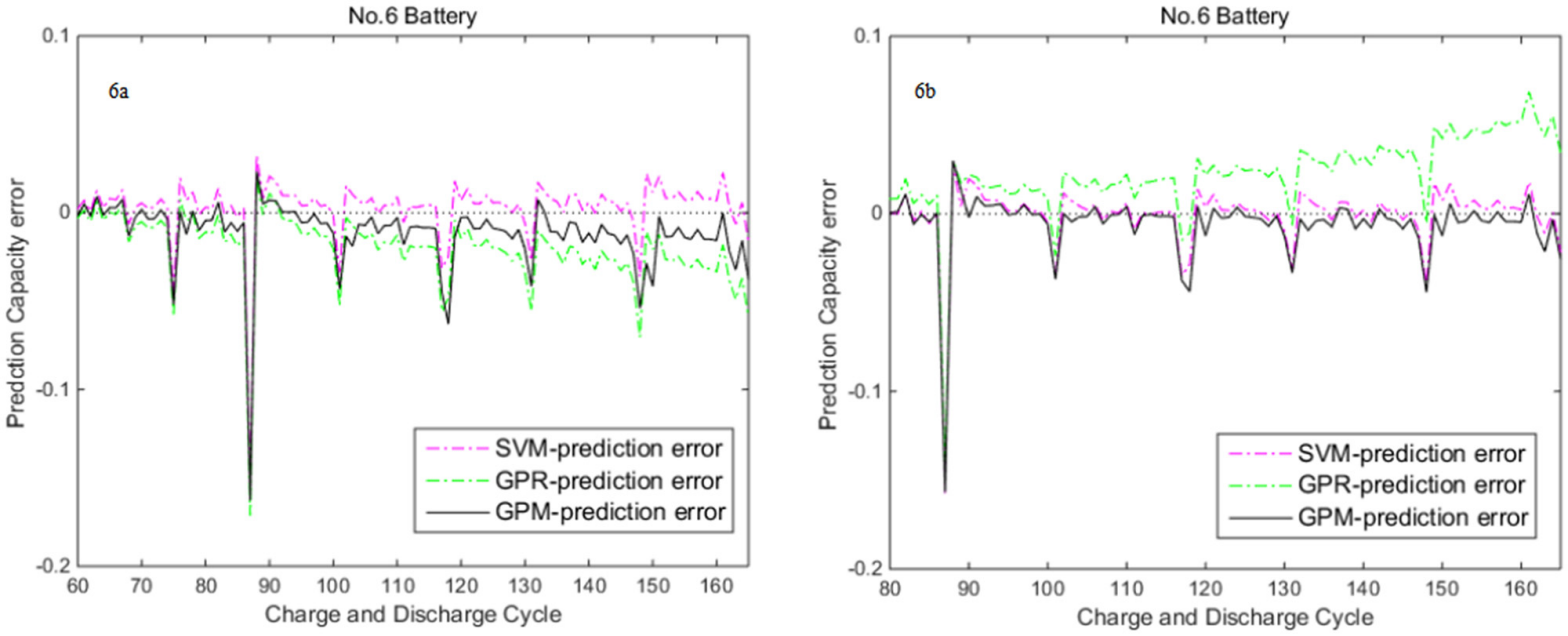
Prediction error curve of No.6 battery with different starting points by three models. (a) the 60^th^ cycle as the starting point. (b) the 80^th^ cycle as the starting point.

In the second part, we choose No.6 battery to predict it’s RUL to demonstrate the GPM’s prediction performance on different batteries. Due to the space limitation, we only demonstrate the prediction errors with different prediction starting points in Fig 6. On the whole, GPM’s prediction performance is still the best among the three kinds of models. Moreover compared Fig 6(a) to 6(b), the up bound of prediction error has decreased 0.02. This is due to the increasing of learning sample by changing the starting point from the 60^th^ cycle to the 80^th^ cycle.

In order to evaluating the prediction performance with more detail by using various models for different batteries, at the same time analyzing the influences of different starting point on predict performance, we compare the RUL prediction between GPM, SVM, and GPR based on No.5 and No.6 batteries respectively. The results are given in Table 1. Consider the RUL values would slightly decreases by using the phase space reconstruction theory to construct training and testing samples, the reconstructed RUL value is selected as the standard comparison object instead of the actual RUL value. This guarantees the accuracy of prediction results comparison.

**Table 1:** Comparison of RUL prediction between GPM, SVM, and GPR of No.5 and No.6 batteries.

Battery number	Starting point of prediction	Prediction model	RUL actul value (Cycle)	Phase space reconstruction parameter	RUL reconstructed value (Cycle)	RUL prediction value (Cycle)	RUL confidence interval of prediction	Capacity of predictive RMSE
**No.5**	No. 60 Cycle	SVM	64	(3,1)	61	62	–	0.0128
		GPR				57	[37,82]	0.0187
		GPM				58	[49,69]	0.0158
	No. 80 Cycle	SVM	44	(3,1)	41	42	–	0.0132
		GPR				44	[32,58]	0.0148
		GPM				43	[40,55]	0.0130
**No.6**	No. 60 Cycle	SVM	48	(3,1)	45	47	–	0.0228
		GPR				41	[19,81]	0.0292
		GPM				43	[34,64]	0.0231
	No. 80 Cycle	SVM	28	(3,1)	25	27	–	0.0227
		GPR				30	[3,55]	0.0336
		GPM				26	[15,44]	0.0207

First of all, comparing three models’ prediction RUL to reconstructed RUL, Table 1 shows that RUL prediction errors of SVM for all four groups are no more than 3 cycles, and GPM model’s RUL prediction errors for all four groups are less than one cycle. However four groups RUL prediction errors of GPR model are up to 5 cycles. Therefore GPM owns higher prediction accuracy based on above comparison. Secondly, we compare the prediction confidence interval of three kinds of models. It is noticed that SVM cannot output the confidence interval. The confidence interval of GPR is wider than the GPM, which means that GPM has higher reliability than GPR in prediction. Finally, we compare the root mean squared error (RMSE) of capacity prediction of three models. It is seen from Table 1 that GPM has the smallest RMSE, and RMSE significantly reduced with the increasing of training samples (that is, the 80^th^ Cycle as the prediction starting point). However the RMSE of GPR and SVM are both bigger than GPM. This verifies that the GPM has higher prediction accuracy than GPR and SVM from another point of view.

## 5. Conclusions

Due to the self-charging and the capacity regeneration, the capacity trajectories of Lithium-ion batteries could be divided into multiple segments with different properties. This multimodality cannot be accurately described by traditional prediction models. Therefore this paper proposed a novel RUL prediction method based on the GPM. It can process multimodality elegantly. Comparative RUL prediction experiments are conducted between GPM, GPR, and SVM based on the NASA datasets of two kinds of commercial and chargeable Type 1850 Lithium-ion batteries. The results illustrate that the GPM method has higher prediction accuracy and confidence. It is noticed that the proposed method is a pure data-driven prediction method. It is not necessary to establish a physical model to depict the degenerate trajectory of battery’s capacity. This kind of appealing feature demonstrates the potential usefulness of the proposed method. Therefore, we believe that our proposed method is a good new tool to the field of the RUL prediction. The future work will emphasize on the study of fast training of GPM model.
